# Access to nicotine products among Polish youth – PolNicoYouth study results

**DOI:** 10.1093/eurpub/ckac131.566

**Published:** 2022-10-25

**Authors:** A Tyrańska-Fobke, Ł Balwicki, D Kaleta, M Goniewicz, G Juszczyk

**Affiliations:** 1 Department of Public Health and Social Medicine, Medical University of Gdańsk, Gdańsk, Poland; 2 Department of Hygiene and Epidemiology, Medical University of Lodz, Łódź, Poland; 3 Department of Health Behavior, Roswell Park Comprehensive Cancer Center, Buffalo, USA; 4 National Institute of Public Health - National Institute of Hygiene, Warsaw, Poland

## Abstract

Smoking and the use of electronic cigarettes pose a risk of cardiovascular disease. The aim of the study was to analyze the availability of these products for Polish youth. The cross-sectional study was carried out in 2020 on a sample of secondary school students (N = 19241) representative of the Polish population, using the CAWI method. In order to estimate the relationship between the independent variables and the outcome variables, the Bayesian multivariate logistic regression was used in the R program using the brms library. It was observed that there were differences in the factors related to the refusal to sell traditional cigarettes and e-cigarettes to Polish youth. The age of adolescents has a more significant relationship (lnBF <2.3) with the refusal to sell them traditional cigarettes (lnBF = 49.65) than e-cigarettes (lnBF = 25.21). Contrary to gender and province of residence, which show a significant relationship only with the refusal to sell e-cigarettes (lnBF = 4.9, lnBF = 3.5). However, they are not related to the refusal to sell traditional cigarettes at all. The amount of expenditure of Polish youth on traditional cigarettes and e-cigarettes significantly depends on the size of pocket money (lnBF = 49.39), the type of school attended by young people (lnBF = 12.19) and the province of residence (lnBF = 3). Other factors, such as age, gender or the size of the place of residence, remain irrelevant. Higher pocket money contributes to higher spending on nicotine products ((lnBF = 12.19). It seems that the age of adolescents does not equally limit access to nicotine products, making e-cigarettes more easily available for sale. Action is needed to effectively limit the access of young people to harmful products.

**Key messages:**

• It seems that the age of adolescents does not equally limit access to nicotine products, making e-cigarettes more easily available for sale.

• Action is needed to effectively limit the access of young people to harmful products.

